# Identification of Peste des Petits Ruminants Virus, Georgia, 2016

**DOI:** 10.3201/eid2408.170334

**Published:** 2018-08

**Authors:** Marina Donduashvili, Ketevan Goginashvili, Natela Toklikishvili, Tamar Tigilauri, Lamara Gelashvili, Lasha Avaliani, Natia Khartskhia, Angelika Loitsch, Arnaud Bataille, Geneviève Libeau, Adama Diallo, William G. Dundon

**Affiliations:** Laboratory of the Ministry of Agriculture, Tbilisi, Georgia (M. Donduashvili, K. Goginashvili, N. Toklikishvili, T. Tigilauri, L. Gelashvili);; National Food Agency, Tbilisi (L. Avaliani, N. Khartskhia);; Austrian Agency for Health and Food Safety, Mödling, Austria (A. Loitsch);; Agricultural Research Center for International Development, Montpellier, France (A. Bataille, G. Libeau, A. Diallo);; International Atomic Energy Agency, Vienna, Austria (W.G. Dundon)

**Keywords:** Georgia, peste des petits ruminants virus, phylogenetic analysis, lineage IV, viruses

## Abstract

A phylogenetic analysis of samples taken from reported outbreaks of peste des petits ruminants virus (PPRV) in Georgia revealed a closer relationship to viruses from northern and eastern Africa than to viruses from countries closer to Georgia. This finding has crucial implications for the control of PPRV in the region.

Peste des petits ruminants virus (PPRV) is the cause of a highly infectious transboundary animal disease that affects primarily sheep, goats, and small wild ruminants. Death rates for PPRV in susceptible animals can be as high as 80% (*1*). Because sheep and goats contribute considerably to the household and cash income and nutrition of small farmers in many countries, the control of PPRV is considered an essential element in the fight for global food security and poverty alleviation. For this reason, PPRV is being targeted by international organizations for global eradication by 2030 (*1*).

Currently, 4 genetic lineages of PPRV are circulating globally. The lineages are defined on the basis of sequence comparison of a fragment of either the nucleocapsid (N) or fusion (F) protein genes of the virus. PPRV lineage IV is found predominantly in Asia and the Middle East, whereas all 4 lineages have been reported in Africa (*2*).

During January–March 2016, outbreaks of PPRV in Tushuri sheep were reported in 3 farms located near Tbilisi, the capital of Georgia. Of 3,740 susceptible sheep, 415 (11%) showed symptoms of PPRV infection, which included necrosis of the commissures of the lips; swelling and bleeding of the gums above the dental pad and buccal mucosa, with white cellular debris on all surfaces, including the tongue; bronchopneumonia (in only a few animals); diarrhea (in 50% of lambs); and loss of appetite. Of the diseased animals, 204 (49%) died, 99 (24%) were humanely destroyed, and the rest recovered. The outbreaks were resolved by the end of March 2016 (*3*). 

Staff of the National Food Agency in Tbilisi collected nasal swabs and ocular samples, which were tested in the laboratory of the Ministry of Agriculture in Tbilisi using a PPR antigen capture ELISA (IDvet, Grabels, France). Six positive samples were individually adsorbed onto the matrix of a ViveST transport tube (ViveBio Scientific, Alpharetta, GA, USA) and were shipped to the Institute for Veterinary Disease Control, Austrian Agency for Health and Food Safety (Mödling, Austria), for further characterization. A part of the same 6 samples was also shipped to the World Organisation for Animal Health reference laboratory at the Agricultural Research Center for International Development (Montpellier, France) for PPRV testing.

In Austria, we eluted the samples from the ViveST with 1 mL of Dulbecco’s modified Eagle medium high-glucose medium and stored them at −80°C. We extracted total RNA from 200 μL aliquots using an RNeasy kit (QIAGEN, Hilden, Germany). We analyzed the extracted RNA samples by reverse transcription PCR (RT-PCR) using the One-Step RT-PCR kit (QIAGEN) to amplify fragments of both the PPRV N and F genes (*4**,**5*). Three of the 6 samples tested were positive by RT-PCR (PPRV/Georgia/G1/2016 [collected January 13, 2016], PPRV/Georgia/G2/2016 [collected February 9, 2016], and PPRV/Georgia/G4/2016 [collected February 9, 2016]). We purified the amplicons and sent them for sequencing using standard Sanger methods at LGC Genomics (Berlin, Germany). The sequences have been deposited in GenBank (accession nos. KY646059–64).

We constructed a phylogenetic tree of N and F gene segments from a representative selection of PPRV sequences available in GenBank, using the maximum-likelihood method available in MEGA6 (https://www.megasoftware.net/*) and* employing the Kimura-2 parameter model of nucleotide substitution with 1,000 bootstrap replications. The phylogenetic analysis revealed that the PPRVs present in the 3 samples from Georgia were identical and belonged to lineage IV (Figure). Of note, the N gene fragment sequences (Figure, panel A) were more related to those of viruses from Egypt, Eritrea, Ethiopia, and Sudan and the F gene fragment sequences clustered with viruses from Egypt, Ethiopia, and Sudan (Figure, panel B). 

**Figure:**
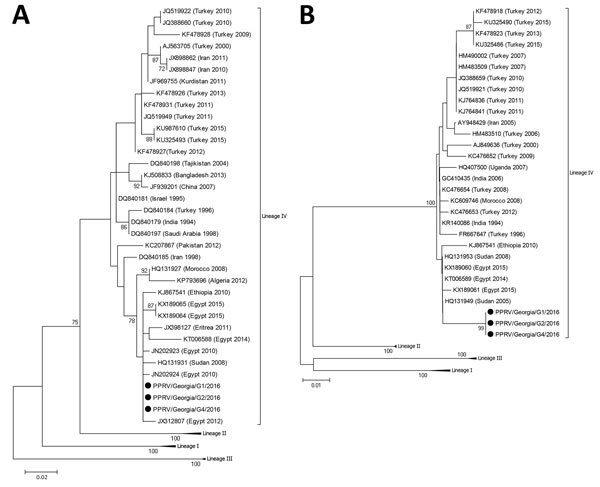
Phylogenetic analysis of peste des petits ruminants virus from Georgia, 2016: A) nucleocapsid (N) gene fragment; B) fusion protein (F) gene fragment. Black dots indicate samples sequenced in this study. Bootstrap values of 1,000 replicates are shown at the nodes. GenBank accession numbers are indicated for reference viruses. Scale bars indicate the number of nucleotide substitutions per site.

Unexpectedly, the N and F gene fragment sequences for viruses isolated from countries close to Georgia (e.g., Turkey, Iran, and Iraq) were less similar to the Georgia viruses than to the ones from Africa. PPRV is a transboundary infectious disease; in many cases, new outbreaks are attributable to incursion from neighboring countries (*6**–**8*). Therefore, a similar situation would have been expected for Georgia, which shares borders with Turkey, Armenia, and Azerbaijan in the south and Russia in the north. Several molecular epidemiologic studies of PPRV lineage IV in Turkey have been performed (*9**–**11*), including more than 200 N and F gene sequence submissions in GenBank covering a period of several years (1996–2015) that provide an up-to-date overview of the PPRVs circulating in the country. However, none of these sequences is similar enough to the sequences from Georgia to indicate a common origin (Figure). To date, Azerbaijan, Armenia, and Russia have not reported PPRV in their countries, which makes it difficult to determine the exact origin of the PPRV identified in Georgia. Because there is no obvious connection between Georgia and Egypt, Eritrea, Ethiopia, or Sudan through the trade or import of small ruminants, further work is required to fully clarify the PPRV situation at a regional level.
